# Rectal, central venous, gastric and bladder pressures versus esophageal pressure for the measurement of cough strength: a prospective clinical comparison

**DOI:** 10.1186/s12931-018-0897-6

**Published:** 2018-10-01

**Authors:** Lluís G. Aguilera, Lluís Gallart, Juan C. Álvarez, Jordi Vallès, Joaquim Gea

**Affiliations:** 1 Department of Anesthesiology, Parc de Salut MAR, Institut Hospital del Mar d’Investigacions Mèdiques (IMIM), Universitat Autònoma de Barcelona (UAB), Passeig Maritim 25, 08003 Barcelona, Spain; 2 Department of Respiratory Medicine, Parc de Salut MAR, Institut Hospital del Mar d’Investigacions Mèdiques (IMIM), Universitat Pompeu Fabra (UPF), CIBERES (ISC III), Barcelona, Spain

**Keywords:** Respiratory muscles [A02.633.567.900], Abdominal muscles [A02.633.567.050], Cough [C23.888.852.293], Laparotomy [E04.406]

## Abstract

**Background:**

Cough pressure, an expression of expiratory muscle strength, is usually measured with esophageal or gastric balloons, but these invasive catheters can be uncomfortable for the patient or their placement impractical. Because pressure in the thorax and abdomen are expected to be similar during a cough, we hypothesized that measurement at other thoracic or abdominal locations might also be similar as well as useful in clinical scenarios. This study aimed to compare cough pressures measured at thoracic and abdominal sites that could serve as alternatives to esophageal pressures (P_es_).

**Methods:**

Nine patients scheduled for laparotomy were asked to cough as forcefully as possible from total lung capacity in supine position. Three cough maneuvers were performed while P_es_ (the gold standard) as well as gastric, central venous, bladder and rectal pressures (P_ga_, P_cv_, P_bl_, and P_rec_, respectively) were measured simultaneously. The intraclass correlation coefficient (ICC) was used to evaluate the repeatability of the measurements in each patient at each site and evaluate agreement between alternative sites (P_ga_, P_cv_, P_bl_, and P_rec_) and P_es_. Bland–Altman plots were used to compare P_es_ and the measurements at the other sites.

**Results:**

Median (first quartile, third quartile) maximum pressures were as follows: P_es_ 112 (89,148), P_ga_ 105 (92,156), P_cv_ 102 (91,149), P_bl_ 118 (93,157), and P_rec_ 103 (88,150) cmH_2_O. The ICCs showed excellent within-site repeatability of the measurements (*p* < 0.001) and excellent agreement between alternative sites and P_es_ (*p* < 0.004). The Bland–Altman plots showed minimal differences between P_es_, P_ga_, P_cv_, and P_rec_. However, P_bl_ was higher than the other pressures in most patients, and the difference between P_es_ and P_bl_ was slightly larger.

**Conclusions:**

Cough pressure can be measured in the esophagus, stomach, superior vena cava or rectum, since their values are similar. It can also be measured in the bladder, although the value will be slightly higher. These results potentially facilitate the assessment of dynamic expiratory muscle strength with fewer invasive catheter placements in most hospitalized patients, thus providing an option that will be particularly useful in those undergoing thoracic or abdominal surgery.

**Trial registration:**

NCT02957045 registered at November 7, 2016. Retrospectively registered.

## Background

Cough is a physiological response whose purpose is to eliminate secretions from the airway tract. Thus, the inability to cough forcefully enough to be effective would increase the risk of pulmonary complications such as atelectasis or pneumonia. This inability is observed in neuromuscular or respiratory diseases and is particularly likely after abdominal or thoracic surgery, when pain, surgical injury and/or the residual effect of anesthetics come into play [1–3].

Because a cough maneuver can be made voluntarily and easily by a patient without training, cough pressure measurements at various sites can be obtained readily in clinical situations, in contrast to the often used maximum mouth expiratory pressure recorded during a static artificial maneuver [4, 5], which must be learned. Cough pressure is usually measured with balloon catheters that record maximum gastric or esophageal pressures (P_ga_, P_es_) [4–6]. It is not always practical to use these catheters outside research scenarios, however, and they can cause discomfort [4, 7]. Furthermore, they cannot be used in certain situations, such as during postoperative recovery from gastrointestinal surgery. Other points of measurement that might potentially be used to reflect cough pressure include central venous pressure (P_cv_), which has been used as an alternative to P_es_ [8]; bladder pressure (P_bl_), which has been compared to P_ga_ [8, 9]; and rectal pressure (P_rec_), which has been used to measure intra-abdominal pressure as an alternative to P_bl_ [10]. However, none of these pressures have yet been used to evaluate expiratory muscle strength, even though many hospitalized patients have a catheter already placed in the superior vena cava or in the bladder.

We hypothesized that these catheters could be suitable for measuring cough pressure without compromising patient comfort, as occurs with the placement of a gastric or esophageal balloon. We also reasoned that placement of a rectal balloon catheter, which does not cause the nausea associated with esophageal or gastric balloons, could also measure cough pressure comfortably. If these hypotheses are correct, wider clinical use of cough pressure to reflect respiratory muscle strength and possible risk of respiratory compromise might be facilitated. The aim of this study was to evaluate the use of P_cv_, P_bl_, and P_rec_ as alternatives to P_es_ or P_ga_ for the measurement of cough pressure.

## Methods

### Patients

Adult patients scheduled for open-midline laparotomy for colon cancer surgery, which required placement of central venous and bladder catheters, were enrolled prospectively. Exclusion criteria included rectal surgery, chronic obstructive pulmonary disease [11], neuromuscular disorders [1], chronic pain, and factors that could impede an adequate recording of the research protocol variables.

The study was approved by the clinical research ethics committee of Parc de Salut Mar (CEIC-Parc de Salut Mar) and by the Spanish Agency for Medicines and Health Products (AEMPS). All patients signed an informed consent form before entering the study, and we provided each with an insurance policy to cover care in the event of adverse events related to the procedures.

### Interventions and measurements

All patients underwent forced spirometry measurement (Datospir 500, SIBEL, Barcelona, Spain) the day before surgery. Reference values were those for a Mediterranean population [11].

Pressures were measured with catheters placed in the esophagus, stomach, superior vena cava, bladder and rectum as follows:

- P_es_ and P_ga_ were measured with compliance balloon catheters (esophageal catheter Jaeger 720,199, Viasys Healthcare, Hoechberg, Germany) as previously described [5]. The catheters were introduced nasally under local anesthesia and the balloons were filled with 1–2 mL of air.
- P_cv_ was measured from the distal port of a double-lumen catheter (CV-26702-E, Arrow, Erding, Germany) placed through the subclavian or internal jugular veins [12]. Correct positioning was checked with the P_cv_ waveform [13].
- P_bl_ was measured with a transurethral (Foley) catheter inserted after the bladder was drained and 50 mL of a 0.9% saline solution was instilled [8, 14, 15].
- P_rec_ was measured with a compliance balloon inserted 10 cm inside the rectum and filled with 5 mL of air [10].

All the pressure curves were displayed on a screen and recorded with a data acquisition system (Acknowledge and MP100, Biopac, Santa Barbara, CA, USA) for off-line analysis. Patients lay in supine position and all the pressure transducers were calibrated and aligned with the axillary midline.

The correct placement of all catheters was assessed by asking the patient to perform a sharp sniff and a cough maneuver while the researcher monitored the signal on the computer screen [5]. Once all catheters were inserted and after a 3-min resting period, baseline respiratory pattern and pressures were recorded.

Cough pressure was then measured at all points. Patients were asked, always by the same researcher (L.G.A.), to cough as forcefully as possible [5] from total lung capacity (TLC). Two or three trials were performed to allow the patient to practice the maneuver before data recording started. Next, the pressures generated by three valid maneuvers, separated by pauses of 5–10 s, were recorded and the difference between the baseline pressure at relaxed end-expiratory lung volume and the peak pressure attained during each cough from TLC was registered, as previously described [4–6]. Maneuvers were considered valid if the patient followed the instruction to cough and the expected cough pressure waveform was observed on the screen [5].

Soon after the end of the protocol, the patients were asked to indicate which catheters caused the least and the most discomfort.

### Statistical analysis

The sample size was calculated to allow us to detect an intraclass correlation coefficient (ICC) value defined as excellent [16] with a statistical power of 80% based on the range of cough pressure values obtained at the different sites during an earlier study [6]. The ICC was used as the measure of the reliability within each patient of the cough pressure measurements at each site and to reflect agreement between alternative sites and P_es_. Bland–Altman plots of mean differences were used to compare P_ga_, P_cv_, P_bl_ and P_rec_ with the gold-standard (P_es_) [17]. P_es_ rather than P_ga_ was chosen as the gold standard because the former is measured in the chest, where a cough effort becomes effective. Maneuvers that generated the maximum P_es_ were chosen for comparison between sites. That maximum value was then compared to the pressures generated by the patient during the same maneuver at each of the other sites.

A *p* value of < 0.05 was considered statistically significant in the ICC analysis. Statistical analysis was performed using IBM SPSS (IBM, Armonk, NY, USA) and STATA (STATA Corp., College Station, TX, USA) software.

## Results

### Participants

Eleven patients initially consented to participate in the study, but one withdrew consent as catheters were about to be inserted. Data from one patient were lost because of technical problems. Thus, we analyzed data for nine patients. Their characteristics are summarized in Table 1. No adverse events were observed during the insertion of the catheters.

**Table 1:** Demographic, anthropometric and functional data

Age (yrs)	66 (53,72)
Gender (male/female)	6/3
ASA class (I/II/III)	0/9/0
Height (cm)	167 (157,169)
Weight (kg)	66 (59,72)
FEV_1_ (L/min)	2.36 (2.2,3.48)
FEV_1_ (% pred)	91 (84,99)
FVC (L)	2.81 (2.73,4.17)
FVC (% pred)	94 (82,97)
FEV_1_/FVC (%)	81 (78,106)

Data are presented as median (first quartile,third quartile) or number of subjects

*ASA* American Society of Anesthesiologists physical status classification system, *FEV*_1_ forced expiratory volume in one second, *FVC* forced vital capacity, *% pred* percentage of the predicted value

### Test results

**Fig. 1:**
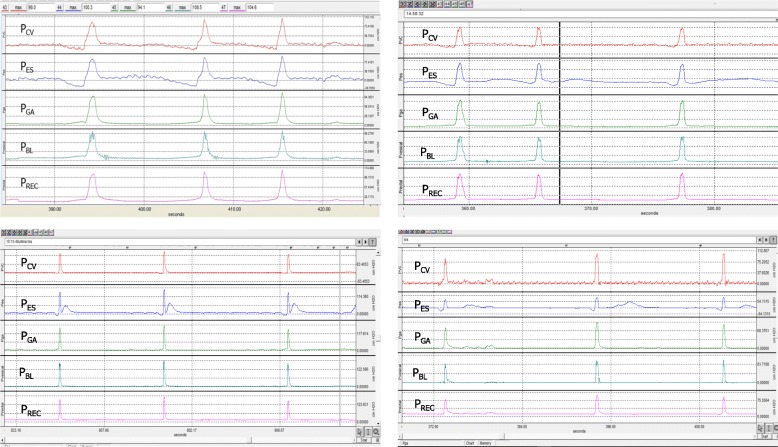
Waveforms at all five measurement sites. Congruent waveforms recorded for four patients at all five measurement sites. P_cv_ = central venous pressure; P_ES_ = esophageal pressure; P_ga_ = gastric pressure; P_BL_ = bladder pressure; P_rec_ = rectal pressure

The cough pressure curves for all sites were congruent. Figure 1 shows the pressure curves for four patients.

The median (first quartile, third quartile) for the maximum P_es_ values for the nine patients was 112 (89,148) cmH_2_O. The other median cough pressures recorded at the same time as the maximum P_es_ values were as follows: P_ga_ 105 (92,156), P_cv_ 102 (91,149), P_bl_ 118 (93,157), and P_rec_ 103 (88,150) cmH_2_O.

Individual measurements recorded for patients at each site are shown in Table 2. Bold-face values show the maneuvers that generated maximum P_es_.

**Table 2:** Measurements of cough pressure at five sites

Patient No.	Maneuver No.	P_es_	P_ga_	P_cv_	P_bl_	P_rec_
1	**1**	**85.3**	**97.4**	**97.6**	**95.9**	**87**
	2	84.6	85.2	93.8	92	82.7
	3	83.2	95	90.9	95.4	88.9
2	1	58.2	52.9	65.8	55.4	51.3
	**2**	**77.3**	**78**	**79.2**	**83.1**	**74.6**
	3	73.8	56.7	72.5	59.6	55.1
3	1	132.8	128.8	127.9	141.1	114.5
	2	137.7	132.5	144.4	153.6	121.5
	**3**	**147.9**	**146.2**	**143.8**	**160.8**	**135.2**
4	1	81.7	82.1	82.3	84.2	81.2
	2	85.5	89.2	95	90.4	88.9
	**3**	**104**	**93.2**	**101.9**	**95.1**	**92.1**
5	1	74.6	70	66.6	78.2	69.3
	**2**	**91.9**	**90.1**	**85.3**	**91**	**89.4**
	3	91.3	83.1	91.2	100.6	82.6
6	1	102.9	101.9	99.8	111.9	100.1
	2	108.3	104.6	105.3	115.5	102.9
	**3**	**112.4**	**105.3**	**101.9**	**117.7**	**102.9**
7	1	110.5	116.5	106.9	128.1	115.9
	**2**	**125.9**	**140.3**	**121.7**	**150.4**	**138.5**
	3	118.5	131.3	114.9	142	131
8	1	148.5	168.8	156.5	167.9	143.3
	2	132.5	152.2	137.7	158	146.9
	**3**	**148.8**	**167.3**	**154**	**152.8**	**162.4**
9	1	121.6	128.4	123.1	137.15	127.2
	**2**	**152.9**	**166.4**	**158.5**	**178.25**	**164.9**
	3	140.9	159.8	143.9	169.65	157.5

Values are in cmH_2_O. Bold-face values identify the maneuver that generated the maximum P_es_

*P*_*es*_ esophageal pressure, *P*_*ga*_ gastric pressure, *P*_*cv*_ central venous pressure, *P*_*bl*_ bladder pressure, *P*_*rec*_ rectal pressure

The ICCs showed excellent repeatability between the three pressures recorded for each patient at each site (within-site repeatability) (*p* < 0.001) and excellent agreement between alternative sites and P_es_ (*p* < 0.004) (Table 3).

**Table 3:** Analysis of cough pressure measurement methods

	ICC	95% CI	*p* value
Within-site repeatability			
P_es_	0.888	0.665–0.972	*p* < 0.001
P_ga_	0.905	0.730–0.976	*p* < 0.001
P_cv_	0.884	0.665–0.971	*p* < 0.001
P_bl_	0.906	0.718–0.976	*p* < 0.001
P_rec_	0.896	0.626–0.975	*p* < 0.001
Between-site agreement			
P_ga_ vs P_es_	0.943	0.784–0.987	*p* < 0.002
P_cv_ vs P_es_	0.974	0.897–0.994	*p* < 0.001
P_bl_ vs P_es_	0.913	0.687–0.979	*p* < 0.003
P_rec_ vs P_es_	0.951	0.811–0.988	*p* < 0.004

*ICC* intraclass correlation coefficient; *CI* confidence interval

**Fig. 2:**
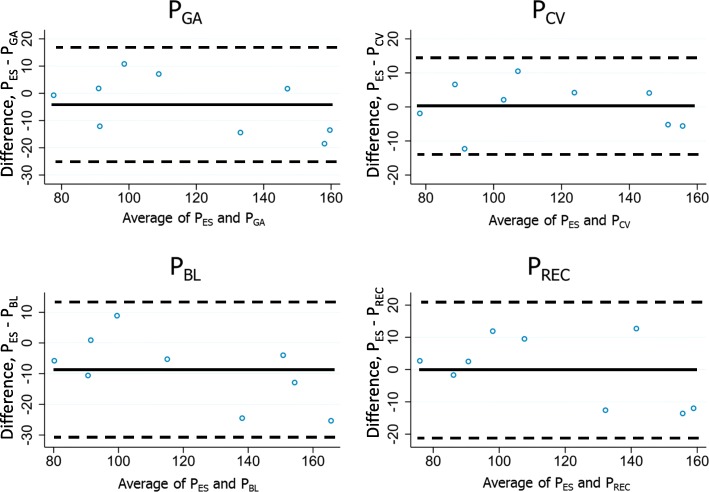
Bland–Altman plots of differences between the maximum (optimal) P_es_ and the pressure measurements at the alternative sites. Circles indicate the difference between PES and the alternative measured pressures. The central line indicates the average difference between the compared measurements. The upper and lower (dotted) lines indicate the 95% confidence interval. Values are expressed in cmH_2_O. P_cv_ = central venous pressure; P_ES_ = esophageal pressure; P_ga_ = gastric pressure; P_BL_ = bladder pressure; P_rec_ = rectal pressure

The median (first quartile, third quartile) differences between maximum P_ES_ and pressures at other sites were as follows: P_ga_ 0.7 (− 1.8,13.5), P_cv_ − 2.1 (− 4.2,5.2), P_bl_ 5.8 (4,12.9), and P_rec_ − 2.5 (− 9.5,12) cmH_2_O. Bland–Altman plots of the differences between P_es_ and each of the other pressures are shown in Fig. 2. P_cv_ and P_rec_ were the alternative-site pressures that showed the best agreement (smallest average differences from P_es_). The average differences between P_bl_ and P_es_ were slightly larger, P_bl_ usually registering higher values than P_es_.

The rectal catheter was the least unpleasant for all the patients, whereas the ones introduced nasally into the esophagus and stomach were the most uncomfortable for seven patients (4 men and 3 women). The bladder catheter was the most uncomfortable for 2 men.

## Discussion

This study demonstrates that cough pressure can be measured with central venous or rectal catheters as alternatives to conventional esophageal balloon catheters. Bladder catheters could also be used although recorded P_bl_ values were systematically slightly higher than the gold-standard P_es_ values.

The ICC analysis indicated excellent repeatability between measurements at the same site, evidence of the precision of cough pressure measurements. In a few patients, cough pressures were slightly lower and differences among repeated maneuvers higher than for the other subjects, but in a series of continuous non-selected patients this minimal variability can be expected. In fact, 5–10% of adult outpatients cannot achieve reproducible pulmonary function results when repeated maneuvers are performed with coaching [18]. The largest source of within-subject variability of spirometry is improper performance of the test [19]. For these reasons, when voluntary maneuvers are recorded several measurements must always be performed and the best one will be chosen.

The ICC analysis also demonstrated excellent agreement between alternative sites and P_es_, and the Bland–Altman plots showed small differences lacking clinical significance between P_es_ and P_ga_, P_cv_ and P_rec_. These data therefore suggest that the alternative measurements are reliably accurate. Thus, P_cv_ or P_rec_ would be valid candidates to choose as surrogates for P_es_. The greater difference between P_bl_ and P_es_, on the other hand, shows that pressure behaves somewhat differently at the bladder, something the clinician would need to bear in mind. Furthermore, given that P_bl_ was usually higher than P_es_, we can conclude that P_bl_ was precise but less accurate than pressure measurements at the other alternative sites. This slight but systematic difference between P_es_ and P_bl_ would mean that the bladder catheter would be the last-choice alternative to the esophageal catheter. P_bl_ could nevertheless be useful in hospitalized patients who already have a bladder catheter in place, so as to avoid disturbing the patient by placing an additional one. The clinician must always take into account this fixed bias in relation to P_es_ when interpreting the pressures.

Although P_ga_ has been used widely to reflect cough pressure in studies of respiratory muscle strength [4–6, 20] and was measured at the same time as P_es_ in this study, we designated P_es_ as the gold standard in the Bland–Altman analysis because it is recorded in the chest, where cough effort takes place. Cough pressures have fluctuated in previous studies of expiratory muscle strength measured with P_es_ or P_ga_ [4, 6, 20–22] because the study populations varied. Higher cough pressures are observed in young, male, and tall subjects as well as in chronic coughers. We measured cough pressure in a specific surgical population, accounting for differences between our results and previously reported values.

Our study was performed under conditions relevant to clinical situations. Patients were in supine position, in which the pressure transducers were all at the same approximate level, favoring reliable comparison between measurement sites. Patients carrying central venous or Foley (bladder) catheters are usually confined to a bed. The cough maneuver was performed from TLC in order to achieve a standardized test measurement [4] and because it is usual to take a deep breath before a cough [5]. Maximum levels of respiratory muscle strength and hence pressure are expected from TLC [5, 23].

The practical implication of our study is that cough pressure can be measured using the technique that best fits the clinical condition of an individual patient. A central venous catheter would be the first choice if one has been inserted. A bladder catheter could also be used provided the clinician bears in mind the larger difference between P_bl_ and P_es_ discussed above. If no catheter has been inserted, a good choice would be a rectal catheter, which our patients found to be the least uncomfortable. P_es_ remains the gold standard, but its main disadvantage, that the insertion of a balloon catheter through the nose causes discomfort [4], was confirmed by our patients. In addition, discomfort can cause esophageal contractions that can impede correct measurement in a considerable percentage of patients [7]. For these reasons, esophageal and gastric balloons should probably be reserved for selected patients or volunteers under experimental conditions.

This study has limitations. The results probably cannot be extrapolated to patients with chronic cough, in whom voluntary cough pressure can be higher than in healthy individuals [20]. In addition, the results possibly cannot be extrapolated to scenarios in which cough is triggered by nerve stimulation [24] or to patient types we excluded. Similar results might well be obtained in these scenarios, but further studies would be needed to confirm that hypothesis.

Our results facilitate further investigation and patient management in many settings. An important scenario is the postoperative period after abdominal or thoracic surgery, where cough effort is reduced [1–3] and where patients are at risk of respiratory complications [25].

## Conclusions

Pressure generated with a cough maneuver from TLC in supine position can be measured in the esophagus, stomach, superior vena cava, or rectum indistinctly. Bladder catheters could also be used, although the recorded pressures would usually be slightly higher than P_es_. These results support assessing expiratory muscle strength for clinical or research purposes without using an additional invasive catheter in most hospitalized patients. If no invasive catheter has already been placed for clinical purposes, a minimally invasive catheter can be chosen for assessing cough pressure.

## Abbreviations

ASA: American Society of Anesthesiologists physical status classification system
CI: Confidence interval
FEV_1_: Forced expiratory volume in one second
FVC: Forced vital capacity
ICC: Intraclass Correlation Coefficient
P_bl_: Bladder pressure
P_cv_: Central venous pressure
P_es_: Esophageal pressure
P_ga_: Gastric pressure
P_rec_: Rectal pressure
TLC: Total lung capacity
